# Genomic basis of adaptation to cardiac glycosides in three insect orders

**DOI:** 10.3389/fgene.2026.1808090

**Published:** 2026-04-22

**Authors:** Kangli Zhu, Chengqi Zhu, Ying Zhen

**Affiliations:** 1 Key Laboratory of Structural Biology of Zhejiang Province, School of Life Sciences, Westlake University, Hangzhou, Zhejiang, China; 2 Westlake Laboratory of Life Sciences and Biomedicine, Hangzhou, Zhejiang, China; 3 Institute of Biology, Westlake Institute for Advanced Study, Hangzhou, Zhejiang, China; 4 Xianghu Laboratory, Hangzhou, China

**Keywords:** adaptation, ATPα, cardiac glycosides, convergent evolution, sodium potassium ATPase

## Abstract

Insects across different orders have convergently evolved adaptations to toxic cardiac glycosides (CGs), which are derived either from their diet or via endogenous synthesis. Previous studies on CG-resistance focused on changes in ATPα that is the direct inhibition target of CGs. Adaptation of whole organisms to toxic CGs could involve orchestrated changes at multiple genes and at multiple biological levels. Here, we explore this possibility by using whole genome sequences to identify several signatures of molecular convergence across multiple CG-adapted species. We identify gene families that changed convergently in CG-adapted species, including one member of stable fatty acyl-CoA reductase, CG5065, carboxylesterases and gustatory receptors that expanded in two of the three species. We find a number of candidate genes under positive selection in all CG-adapted species. We also identify convergent amino acid substitutions that have independently evolved in CG-adapted insects, including a conserved gene involved in the septate junction, Gliotactin (*Gli*). We used CRISPR–Cas9 to generate viable, homozygous *Gli* knock-in *Drosophila* lines with the convergent substitution. Through egg-larva and larva-adult survival experiments, we found that mutant flies consistently exhibit a lower survival rate compared to wild-type lines. Transmission electron microscopy (TEM) analysis of stage 17 embryos showed that in Gli mutants, the dihedral angles of bicellular membranes near the tricellular junction (TCJ) were unequal, and electron-dense materials were absent in the TCJ center. We propose that this convergently evolved *Gli* variant may contribute to CG adaptation by modulating epithelial permeability, potentially facilitating the sequestration of toxic CGs.

## Introduction

Convergent evolution is the independent evolution of similar traits across distantly related lineages, usually as results of similar selective pressure or adaptation to similar ecological niches. Dissecting the molecular basis underlying convergent traits across multiple lineages allows one to assess the extent to which adaptive phenotypic changes are caused by the recruitment of the same genes or even same mutations.

The evolution of cardiac glycoside (CG) resistance in insects represents a classic case of convergent adaptation. CGs, produced by plants and some insects as defense compounds, inhibit the Na+/K + -ATPase (sodium pump) by binding to its α-subunit (ATPα), a highly conserved enzyme critical for neural and muscular functions (Smith, 1989; Wasserstrom and Aistrup, 2005). Despite this toxicity, multiple insect lineages across 300+ million years of evolution have independently adapted to CG-rich diets (Agrawal et al., 2012; Dobler et al., 2015; López-Goldar et al., 2022; Agrawal and Hastings, 2023). Notable examples include the monarch butterfly (*Danaus plexippus*) and large milkweed bug (*Oncopeltus fasciatus*), which specialize on cardenolide-producing milkweeds and sequester these toxins for predator defense (Brower and Glazier, 1975; Chipman, 2017; Jones et al., 2019; Agrawal et al., 2023). Moreover, some fireflies such as common eastern firefly (*Photinus pyralis*) can *de novo* synthesize lucibufagins, also a class of CGs (Gao et al., 2011), which are used in defense and mating (Branham and Wenzel, 2003; Lewis and Cratsley, 2008). The lifestyle and the context of CGs utilization and exposure differ dramatically between fireflies and herbivores, so species-specific adaptation mechanisms are expected. From this perspective, monarch butterfly and large milkweed bug may share more similarity because of their phytophagous lifestyle. Nevertheless, all three species share the ability to tolerate high level of CGs in their body, thus they may share some common mechanisms that facilitate CGs adaptation.

As the direct target of CGs, ATPα has been the focus of most studies on CG resistance due to its role in this convergently evolved trait. Research has revealed striking molecular parallels in ATPα across CG-resistant herbivorous insects from different orders, including recurrent amino acid substitutions, gene duplications, and regulatory changes leading to similar tissue-specific expression patterns—all contributing to the evolution of CG resistance (Dobler et al., 2012; Zhen et al., 2012; Yang et al., 2019; Herbertz et al., 2023; Yang et al., 2023).

In addition to modifications in ATPα, other mechanisms may contribute to CG insensitivity and adaptation, though they are less well understood. Potential alternative mechanisms include altered permeability barriers that prevent CG entry into sensitive tissues or sequester toxins into insensitive compartments. For example, the red beetle (*Lilioceris merdigera*) excretes nearly all ingested ouabain and digoxin, indicating a strong gut barrier (Baum and Dobler, 2023). Similarly, the midgut and septate junctions in the blood-brain barrier of *Manduca sexta* may block polar CGs from entering neural tissues (Barbehenn, 2001; Petschenka et al., 2013). For nonpolar CGs like digoxin, which can passively cross membranes, active efflux transporters such as multidrug resistance proteins (MDRs), P-glycoproteins (P-gps), and organic anion transporting polypeptides (OATPs) may counteract diffusion. Knockdown of OATP or MDR genes increases susceptibility to CGs in the rice brown planthopper (*Nilaparvata lugens*) (Li et al., 2025), and *Drosophila* mutants lacking these transporters show greater neurological damage on high-cardenolide diets (Groen et al., 2017), highlighting their protective role against dietary CGs.

Metabolic detoxification is another key mechanism insects use to adapt to xenobiotics, involving a multi-phase process where lipophilic compounds are enzymatically converted into water-soluble metabolites for excretion (Dermauw and Van Leeuwen, 2014; Koirala et al., 2022; Cruse et al., 2023; Wu et al., 2025). Cytochrome P450s (CYP450s) and carboxylesterases (COEs) initiate this process by transforming xenobiotics into more hydrophilic forms (Yu et al., 2009; Montella et al., 2012; Feng et al., 2018; Cruse et al., 2023). Subsequently, glutathione-S-transferases (GSTs) and UDP-glycosyltransferases (UGTs) conjugate these detoxified products, enabling ATP-binding cassette (ABC) transporters to expel them from the cell (Dermauw and Van Leeuwen, 2014; Amezian et al., 2021; Koirala B K et al., 2022).

Although some of these mechanisms have been proposed to confer adaptation to CGs, previous studies focused on limited number of candidate genes and at most a couple of species. Adaptation to high levels of CGs may involve the above aspects of adaptation in addition to changes in ATPα, including but not limited to changes in permeability of the diffusion barriers, specialized transporters for tissue-specific enrichment or exclusion of CGs, and enzymatic detoxification.

Here we take advantage of the recent availability of three high quality genomes of CG-adapted species, *i.e.*, monarch butterfly, large milkweed bug and common eastern firefly, to identify genome-wide molecular signatures of convergence from three perspectives, including convergent gene family expansion and contraction, convergent positive selection and convergent amino acid substitutions in coding genes. The goal of this unbiased genome-wise approach is to identify new candidate genes and mechanisms that contribute to CG adaptation that are shared by both herbivorous species that sequester CG toxins from host plant and fireflies that *de novo* synthesize the CG toxins themselves. We also use transcriptome data from subclade of species to reproduce the detected enrichment pattern, and to explore the potential of utilizing the more available yet more incomplete and error-prone transcriptome data to do similar kind of scan for molecular convergence.

## Materials and methods

### Genomic data and orthogroup identification

For each of the 15 species, we downloaded the annotated coding sequences (CDS) directly from the NCBI RefSeq database (accession numbers listed in Supplementary Table S1). Specifically, we retrieved the cds_from_genomic.fna files, which contain the nucleotide sequences of predicted protein-coding regions extracted from the respective whole-genome assemblies. These sequences represent spliced exons (introns removed) as defined by the official NCBI genome annotations. Additionally, the corresponding translated protein sequences (translated_cds.faa) were obtained, we chose the longest isoform, and used OrthoFinder (Emms and Kelly, 2019) to infer orthogroups with default parameters.

In order to increase the number of orthogroups included in the scan of amino acid convergence, we used UPhO pipeline (git version 4ec1589) (Ballesteros and Hormiga, 2016) to delineate the orthogroups containing multiple gene copies in the outgroup species. Amino acid sequences for each orthogroup were aligned with MAFFT (v7.407) (Katoh and Standley, 2013). Poorly-aligned regions were trimmed by TrimAl v1.4 (Capella-Gutierrez et al., 2009), and sequences with extensive gaps and missing data were removed by Al2Phylo using parameter setting “-m 20 -p 0.30 -t 2”. Specifically, these settings removed sequences shorter than 20 residues, retained only sequences with at least 30% non-gap coverage, and ensured that the final cleaned alignments contained a minimum of two taxa. For each orthogroup, the maximum likelihood tree was built using the default settings of FastTree (v2.1.11) (Poon et al., 2010). UPhO.py script was used to delineate from clades with a bootstrap value≥0.95, with at least four species represented and “in-paralogs” allowed. After the delineation, the orthogroups containing exactly one gene copy in three target species and at least two outgroups were retained for following selective pressure and convergence analyses (referred to as single copy genes thereafter).

### Gene family expansion and contraction

To identify orthogroups that experience convergent pattern of expansion or contraction in three target CG-adapted species, we used CAFE pipeline (De Bie et al., 2006) to examine the pattern of gene family evolution in the 15-species full genome dataset. Species tree was from (Misof et al., 2014; Zhang et al., 2018; Kawahara et al., 2019), and divergence time was obtained from TimeTree database (http://www.timetree.org/).

We excluded orthogroups with more than 100 members following CAFE’s manual, since families with high gene copy number variation can lead to non-informative parameter estimates. In order to account for assembly errors, we estimated an error parameter with a single lambda of gene birth and death. Then we re-estimated the birth and death rates separately and identified rapidly evolved families for each terminal branch. A conditional *p*-value was calculated for each gene family, and only families with *p* < 0.01 were considered. We also allowed three CG-adapted species to have a different lambda (*i.e.*, two lambda model). Two lambda model was compared to single lambda model by performing a likelihood ratio test according to the CAFE’s manual.

### Detection of positive selection

Coding sequences from single copy genes were used for selective pressure analyses. Sequences with premature stop codons were excluded. We aligned these coding sequences and removed poorly align regions with TranslatorX (Abascal et al., 2010). Briefly, TranslatorX translates the coding sequences to amino acid sequences using the standard genetic code, aligns the sequences with PRANK (v.170,427) (Loytynoja and Goldman, 2008), filters the alignments with GBlocks 0.91b (Talavera et al., 2007) with the following parameters “- b1=(n_seq*0.75 + 1) -b2 = n_seq*0.85 -b3 = 2 -b4 = 5 -b5 = h”, and then back-translates them to nucleotide sequences. We further excluded sequences with 90% missing data, and manually checked several alignments to confirm their accuracy. Gene trees were built with RAxML (Stamatakis, 2006) using the GTRGAMMA model, and they were used for the following selective pressure and convergence analyses.

The influence of natural selection acting on protein-coding genes was inferred by estimating the ratio of the nonsynonymous and synonymous substitutions (ω). In brief, ω > 1, = 1 and <1 indicate positive selection, neutral evolution and negative selection, respectively. We used program *codeml* for selective pressure analyses in PAML (v4.9i) (Yang, 2007). Specifically, we used branch-site model (Ma vs. Ma1) to detect signals of positive selection in three CG-adapted insects, setting them as foreground lineages. Alternatively, we detected genes under positive selection in each CG-adapted species by setting only one CG-adapted species as foreground lineage and excluding two other CG-adapted species. The *p*-values were estimated assuming a null-distribution that is a 50:50 mixture of a χ^2^ distribution and a point mass at zero (Yang, 2007). The *p*-values were corrected for multiple testing, and a false discovery rate (FDR) cutoff of 0.05 was used. Since branch-site test is highly sensitive to errors in sequence alignment (Wong et al., 2008; Mallick et al., 2009; Privman et al., 2012), we not only used Gblocks to mask poorly aligned regions (Talavera et al., 2007), but also manually checked the sequence alignment for each positively selected gene and removed the genes if positively selected sites were distributed within the ambiguous regions.

### Detection of convergent amino acid substitutions

Coding sequences from single copy genes were used for detection of amino acid convergence. We used four methods to detect convergent signals among three CG-adapted insects. In “identical method” (Zhang and Kumar, 1997), a substitution is defined as convergent if it changes from same or different ancestral states to the exact same derived amino acid in different lineages. The ancestral states were extracted from PAML output, and we required convergent substitutions to be exclusively shared by CG-adapted insects.

### Gene enrichment analyses

We used topGO (Alexa et al., 2006) to do the gene enrichment test, and four algorithms were integrated in topGO. *Classical* method has higher power and sensitivity, with high false-positive rate that is introduced by the dependencies between top scoring nodes. *Elim*, *weight* and *parent-child* methods use different approaches to reduce the dependencies between the individual term’s measurements, and therefore may have better performance in reducing false positive rate (Alexa et al., 2006; Grossmann et al., 2007). The tested lists were genes harboring convergent substitutions, positively selected genes and rapidly evolving gene families in CG-adapted lineages. The background gene list was customized to all single copy genes.

### Transcriptome data and *de novo* assembly

RNAseq data of ten CG-adapted species and nine outgroups from three insect orders were obtained from two previous studies (Zhen et al., 2012; Sander and Hall, 2015) (Supplementary Table S2; Supplementary Figure S1). Transcriptome for each species was assembled using Trinity (v2.8.4) (Grabherr et al., 2011; Haas et al., 2013) with option-trimmomatic. Transdecoder (v5.5.0) in the Trinity package was used to identify putative coding regions with default parameters. Coding regions of *Photuris frontalis* were obtained directly from (Sander and Hall, 2015). The longest isoform of the predicted peptides for each gene was used in following analyses. Sequences of species from Lampyridae were obtained from (Zhu et al., 2024).

We used OrthoFinder (v2.3.1) (Emms and Kelly, 2019) to identify orthogroups from peptide sequences predicted from the above 19 transcriptomes and eight species with full genomes (Supplementary Table S2; Supplementary Figure S1). UPhO pipeline (Ballesteros and Hormiga, 2016) was used to delineate orthogroups containing multiple gene copies in outgroup species.

### Generation of the Gli V581L site-directed mutant

We generated the site-directed mutation V581L in *Gli* to test its function in CG adaptation. The CRISPR/Cas9-mediated targeted mutagenesis of *Gli* (CG3903) from w1118 was performed by Fungene Biotech (http://www.fgbiotech.com), following primarily previously published procedures (Bassett et al., 2013). Briefly, sgRNA (synthetic guide RNA) targets were designed with CHOPCHOP (http://chopchop.cbu.uib.no/) (Montague et al., 2014; Labun et al., 2016; Labun et al., 2019). Two sgRNAs were designed: CCA​CTG​TGG​AGG​CAC​TGA​ATC​TG and GTA​CGT​ACT​GAA​CAC​CAC​TGT​GG. *In vitro* transcription of sgRNAs was performed with the Megascript T7 Kit, and Cas9-mRNA was produced by *in vitro* transcription of optimized Cas9-DNA plasmid sequence with the use of the mMESSAGEmMACHINE T7 kit. For donor vector construction, a 2.7 kb *Gli* gene region containing the site-directed mutation was cloned into pBluescript SK (−) vector. Then, they injected mixture of sgRNA-Cas9-mRNA-donor into 400–500 eggs of w1118 fly line, and PCR sequencing was performed to identify whether the offspring flies carried the designed mutation.

### Hatching rate and feeding experiments in wild-type and site-directed flies

To see the potential impact of V581L mutation in *Gli*, we conducted a series of experiments to see its influence on behaviors of wild type (w1118) and knock-in (V581L) flies. Briefly, to assess early larval survival, eggs were collected within 2 h of laying and 50 individuals from each line were introduced into vials containing standard medium supplemented with a series of ouabain concentrations. Ouabain (>95 purity) was obtained from MCE (CAS No:11018–89–6), and was administered at concentrations of 0, 1, 5, and 9 mM. The number of second-instar larvae was recorded 48 h post-oviposition after rearing at 25 °C (consistent with standard *Drosophila* developmental conditions). Each treatment group was tested in three independent replicate vials. For feeding experiments, we introduced 50 second-instar larvae in wide vials containing standard medium supplemented with a series of ouabain concentrations. Pupariation, adult eclosion, and survival were recorded over a period of ∼21 days until all adult flies had eclosed.

### Embryonic electron microscopy analyses

To further investigate the potential effects of the *Gli* mutation on septate junction, we employed transmission electron microscopy (TEM) to examine the ultrastructure of stage 17 wild-type and the mutant embryos. A total of nine eggs from each group, wild-type and Gli mutant, were prepared for TEM analysis. To eliminate artefacts, we applied high-pressure freezing–freeze substitution (HPF–FS) to fix the eggs. After fixation, embryos were sectioned and imaged in a 120 kv transmission electron microscope.

## Results

### Identification of orthologous genes from whole genomes

**FIGURE 1:**
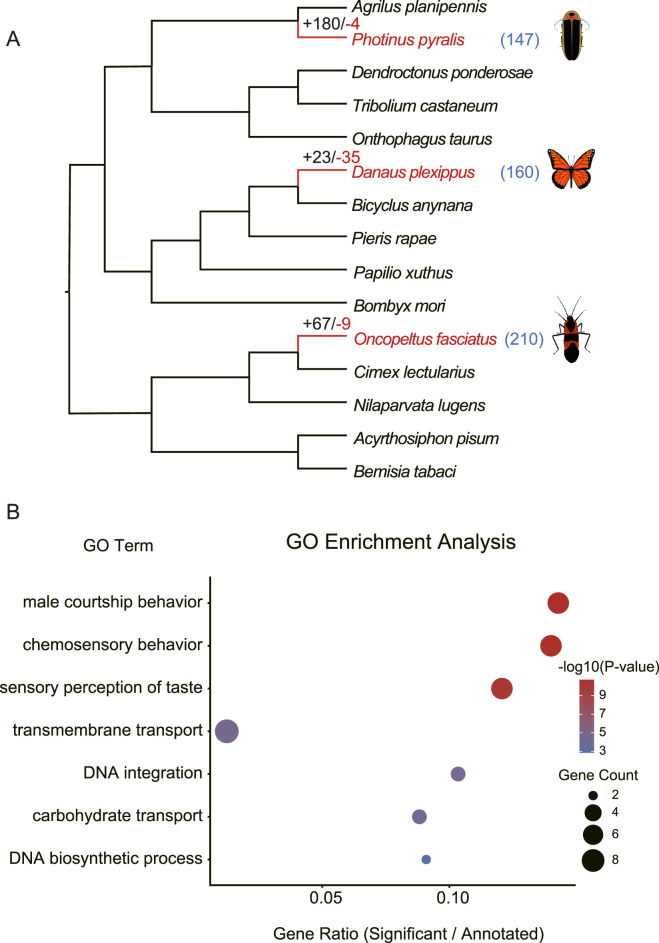
Phylogenetic relationships and evolutionary dynamics of gene families in studied species. **(A)** Phylogenetic tree of the species included in our analyses. Red branches denote cardiac glycoside (CG)-adapted lineages. Numbers adjacent to each branch indicate the counts of specific evolutionary events: gene loss (red), gene duplication (black), and genes under positive selection (blue). **(B)** Gene Ontology (GO) enrichment analysis of rapidly evolving gene families shared by at least two CG-adapted species. The plot displays the top enriched terms, with dot size representing the number of genes and color intensity indicating statistical significance.

To identify genome-wide signatures of molecular convergence underlying CG-adaptation, we compiled protein coding sequences from three CG-adapted insect species and 12 outgroup species with full genomes sequenced (Figure 1A; Supplementary Table S1) (Misof et al., 2014; Zhang et al., 2018; Kawahara et al., 2019). We consider this dataset to include complete coding gene sets given relatively high-quality genomes. In this dataset, three insect orders each has one representative CG-adapted species, including monarch butterfly in Lepidoptera, large milkweed bug in Hemiptera and common eastern firefly in Coleoptera. We identified a total of 13,762 orthogroups among these 15 species using OrthoFinder (Emms and Kelly, 2019).

### Gene family evolution in CG-adapted species

To detect convergent patterns of gene family expansion and contraction that could contribute to adaptation to toxic CGs, we used CAFE (De Bie et al., 2006) to identify rapidly evolving gene families in three CG-adapted species. Certain gene families have high rates of gene birth and death dynamics in multiple insect lineages, and true ortholog identification for large families could have high error rates (Oakeshott et al., 2005; Suzuki et al., 2018; Dermauw et al., 2020). Thus we excluded orthogroups with more than 100 members from all analysis according to CAFE’s manual. Secondly, assembly errors of a genome can lead to deviation of observed gene copies numbers from the true ones, so we account for the assembly errors using an error model, with a single rate (lambda) of gene birth and death. The global error rate is estimated to be 0.0125, and the gene birth/death rates (lambda) for error model and no error model are the same (lamda = 0.0011). Lastly, we tested both single lambda model and two lambda model that allows three CG-adapted species and outgroups to have different lambda values, and found that two lambda model fits the observed data significantly better than single lambda model (*p* < 0.01). The number of rapidly evolving families using two lambda model is 58 (monarch butterfly), 184 (common eastern firefly) and 76 (large milkweed bug), respectively (Figure 1A).

Among the rapidly evolving gene families, none has same pattern of expansion or contraction in all three target species. 21 gene families have convergent patterns of expansion in two of the three CG-adapted insects, while none contracted in more than one target species (Supplementary Table S3). To investigate functions that rapidly evolving genes may play a role in, we used topGO to do the functional enrichment analyses (Alexa et al., 2006; Grossmann et al., 2007). Analysis of these 21 convergently expanded families revealed that chemosensory behavior (GO:0007635) and sensory perception of taste (GO:0050909) were among the most significantly enriched terms (Figure 1B; Supplementary Table S4). Notably, this set included a gustatory receptor family homologous to *Drosophila melanogaster* gustatory receptor 28 (GR28), which is known to function in chemosensory perception. When analyzing rapidly evolving gene families within each species independently, we found that these same chemosensory and taste perception terms were also significantly enriched in both the large milkweed bug and the common eastern firefly (Supplementary Table S4). Beyond chemosensation, α-esterases involved in detoxification also expanded in both large milkweed bug and common eastern firefly (Supplementary Table S3). We also found that four gene families expanded in both monarch butterfly and large milkweed bug, and one of these is homologous to *D. melanogaster* fatty acyl-CoA reductase (FAR) CG5065.

### Positively selected genes in CG-adapted species

To identify genes under positive selection and amino acid substitutions that evolve convergently in three CG-adapted species, we used 4,092 orthogroups that contain exactly one gene copy in every target species and at least two outgroups (see Methods). We used branch-site model (Ma vs. Ma1) in PAML (Zhang, 2005; Yang and dos Reis, 2010) to identify positively selected genes (PSGs) in CG-adapted insects. Since branch-site test is highly sensitive to errors in sequence alignment (Wong et al., 2008; Mallick et al., 2009; Privman et al., 2012), in addition to removing poorly aligned regions systematically using Gblocks (Talavera et al., 2007), we also manually checked the alignments for all identified PSGs and removed positively selected sites in ambiguously aligned regions. We aim to identify candidate genes under positive selection in all three target species, without requiring the exact same sites to be under selection, thus two approaches were taken. First, in one test we set all three CG-adapted species as foreground, and found 94 genes under positive selection (Supplementary Table S5). Secondly, we conducted three tests, each setting one target species as foreground and excluding the other two species. We find 160, 210 and 147 PSGs in the branch leading to monarch butterfly, large milkweed bug and common eastern firefly, respectively (Figure 1A). Two of these PSGs were shared by all three CG-adapted lineages, and they are annotated as chromodomain-helicase-DNA-binding protein 7 (*Chd7*) and vitellogenin receptor (*Vgr*) (Supplementary Table S5). A total of 22 PSGs were exclusively shared by herbivorous monarch and butterfly including crumbs (*crb*) and lethal (2) giant larvae protein (*l(2)gl*) (Supplementary Table S5). Enrichment analysis of positively selected genes yielded diverse functional categories with no clear convergence on toxin-resistance pathways (Supplementary Table S6).

### Convergent amino acid substitutions in CG-adapted species

To detect convergent amino acid substitutions in CG-adapted species, we applied identical method to look for the exact same amino acid substitutions in clades with convergent phenotypes (Zhang and Kumar, 1997). Using identical method, we found 23 convergent amino acids in 23 genes shared by all three CG-adapted species (Supplementary Table S7), and 1,738 amino acids in 1,215 genes converged in two of three CG-adapted species. Considering the difference in lifestyle and the context of CGs utilization and exposure between fireflies and herbivores, we found 386 amino acids in 336 genes converged in two herbivores, i.e., large milkweed bug and monarch butterfly.

**FIGURE 2:**
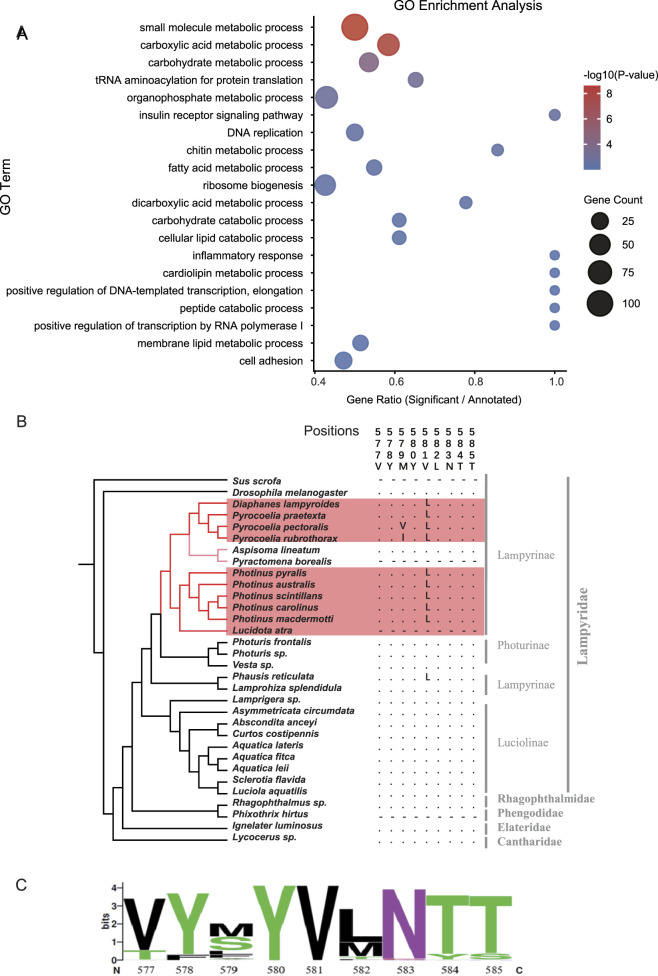
Convergent amino acid substitutions and the identification of the *Gli* adaptive mutation. **(A)** Gene Ontology (GO) enrichment analysis of genes harboring identical convergent amino acid substitutions in at least two CG-adapted species. The plot highlights significantly enriched biological processes, dot size represents the number of genes, and color indicates statistical significance. **(B)** Amino acid sequences of Gli from representative species across Lampyridae and other lineages. Nearly all CG-resistant species possess the V581L substitution (Valine to Leucine at position 581). **(C)** WebLogo analysis of the protein region surrounding site 581, based on sequence alignments from EnsemblMetazoa. The logo demonstrates that Valine (V) is highly conserved at this position across the vast majority of insect species.

To investigate functions that convergent genes may play a role in, we used topGO to do the functional enrichment analyses (Alexa et al., 2006; Grossmann et al., 2007). Among the top 20 enriched terms for genes harboring identical convergent sites, cell adhesion (GO:0007155) was significantly enriched in the CG-adapted species (Figure 2A; Supplementary Table S8). Furthermore, when restricting the analysis to genes with convergent sites shared by at least two species, this term remained significantly enriched, specifically driven by the convergence between the large milkweed bug and the common eastern firefly (Supplementary Figure S2; Supplementary Table S8). Based on the significant enrichment of cell adhesion, we manually curated a list of 25 convergent genes. Our selection criteria extended beyond strict GO term membership to include genes with established roles in cell-cell interaction, and epithelial barrier function as supported by literature, ensuring a comprehensive capture of the adaptive mechanism. For example, five sites in five genes were shared by herbivorous large milkweed bug and monarch butterfly, including S/V227N in flapwing (*flw*), T162S in shaggy (*sgg*), A/I451T in Neurexin IV (*Nrx-IV*), D1269E in starry night (*stan*) and V/S165N in Src oncogene at 64B (*Src64B*) (Supplementary Table S9). In Macroglobulin complement-related (*Mcr*), V395I substitution is shared by all three CG-adapted insects, and D412N was shared by both large milkweed bug and common eastern firefly.

To examine whether these convergent substitutions were shared by more CG-adapted species, we took advantage of transcriptome data that are available for more taxa. We identified orthologs of the above 25 candidate genes from assembled transcriptomes of ten more CG-adapted species and nine outgroups. We found that substitution D412N in *Mcr* is also present in firefly *P. frontalis*, a predator that can sequester CGs by feeding on *Photinus* (Eisner et al., 1997), and small milkweed bug (*Lygaeus kalmii*), a close relative of large milkweed bug. Moreover, we identified a highly conserved substitution, V581L in *Gli*, shared by the large milkweed bug and the common eastern firefly, which is also present in the CG-adapted small milkweed bug. By adding more beetles from Lampyridae, we find that almost all CG-resistant species contain the V581L substitution (Figure 2B). The valine at this position is exceptionally conserved across Metazoa, being identical in all insecta species with reported *Gli* orthologs from EnsemblMetazoa (Figure 2C), suggesting that the V581L substitution may have significant functional implications.

### Survival rate was affected in the Gli mutant flies

**FIGURE 3:**
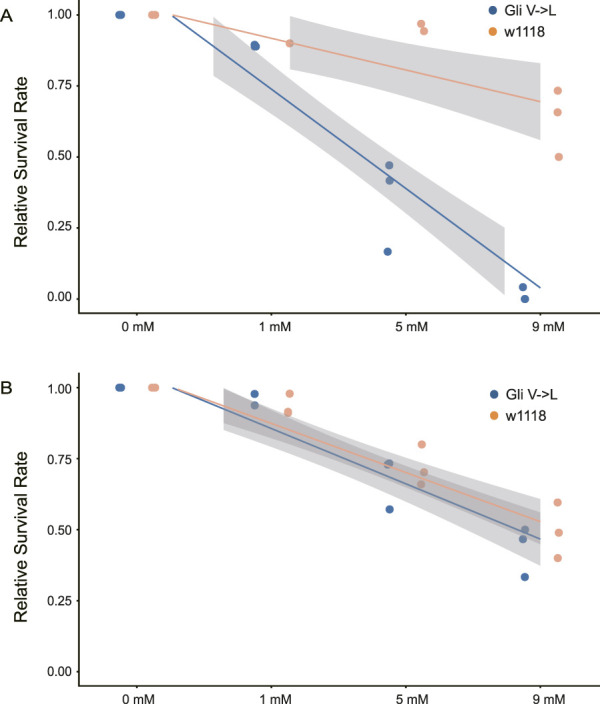
Relative survival rates analyses in wild-type and *Gli* mutant *Drosophila*. Survival rates of *Gli* mutants and wild-type controls from egg to second-instar larva (48 h) **(A)** and second-instar larva to adult **(B)** across varying ouabain concentrations. For each replicate, survival values were normalized to the 0 mM control condition to correct for baseline differences among lines. This normalization allows for a direct comparison of dose-response sensitivity across genotypes. The orange line represents the survival rate of wild-type flies, while the blue line represents the survival rate of *Gli* mutant flies.

To validate the prediction results of our convergence scan, we focused on the conserved convergent substitution in *Gli* (V581L). Since *Gli* mutations result in an incomplete peripheral blood-nerve barrier in the peripheral glia of *D. melanogaster* embryos, leading to major physiological and behavioral deficits (Auld et al., 1995). To assess whether mutations in the *Gli* gene can affect behavioral performance, we used CRISPR–Cas9 genome editing to generate viable, homozygous *Gli* knock-in *Drosophila* lines carrying a precise substitution at site 581 (V581L). Through egg to second-instar larva and second-instar larva to adult survival experiments, we found that mutant flies consistently exhibit a lower survival rate compared to wild-type lines (Figure 3). The primary difference is that, for wild-type flies, egg-larva survival gradually declined as concentrations increased, whereas for mutants, the egg-larva survival rate dropped sharply with increasing concentrations (Figure 3A). Additionally, larva-adult survival decreased with increasing concentration for both wild-type and mutant flies (Figure 3B).

### Gli is required for tricellular junctions

**FIGURE 4:**
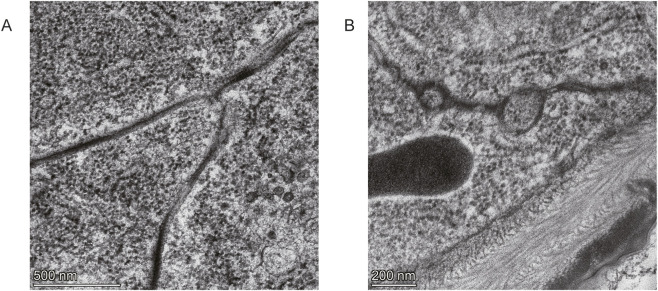
Transmission electron micrographs of embryos from wild-type and *Gli* mutant *Drosophila*. **(A)** Septate junctions in stage 17 embryos of *Gli* mutant flies **(B)** Septate junctions in stage 17 embryos of wild-type flies.

Because *Gli* is a novel marker of tricellular junctions and is necessary for septate junction development (Schulte et al., 2003), we analyzed junction integrity in stage 17 *Gli* mutant and wild type *Drosophila* embryos by TEM. For both wild-type and *Gli* mutant embryos, two to three replicates were analyzed, and their ultra-structures were consistent. In wild-type embryos, the dihedral angles of bicellular membranes are equal (∼120°) near the tricellular junction (TCJ). In *Gli* mutants, however, these angles are unequal (Figure 4). Additionally, electron-dense materials are present in the bicellular septa near the TCJs in wild-type embryos but are absent from the TCJ center in Gli mutants (Figure 4). For wild-type and Gli mutant embryos, we obtained two to three replicates and their ultrastructures are the same. Similar abnormalities in the TCJ caused by the Gli mutation were observed in aka mutant *Drosophila* embryos, which are also crucial for epithelial barrier function and tricellular junction assembly (Byri et al., 2015).

## Discussion

In this study we take an unbiased approach to identify multiple genomic signatures of molecular convergence across multiple lineages of insects that independently evolved adaptation to toxic CGs. We find gene families with convergent pattern of expansion or contraction, genes under convergent positive selection, and genes that harbor convergent amino acid changes in CG-adapted species. We utilized CRISPR–Cas9 to generate viable, homozygous *Gli* knock-in *Drosophila* lines with the convergent substitution. TEM analysis of stage 17 embryos revealed abnormal tricellular junction structures in the mutant Gli flies.

### Rapidly evolving gene families in CG-adapted insects

Resistance to plant-derived or anthropogenic toxins could be achieved by specific changes in target proteins and detoxification of toxins. Known detoxification enzymes include CYPs, GSTs and COEs (Dermauw and Van Leeuwen, 2014; Dermauw et al., 2020; Koirala et al., 2022; Cruse et al., 2023; Wu et al., 2025). Insect COEs hydrolyze a wide range of xenobiotics, including pesticides, insect and plant odors, pheromones, hormones, and environmental toxicants (Oakeshott et al., 2005; Montella et al., 2012; Cruse et al., 2023). Their versatile hydrolytic capabilities play crucial roles in pesticide resistance and host plant adaptation. The COEs can be divided into different clades in insects, including α-esterases that are intracellular catalytically active enzymes and play important roles in dietary/xenobiotic detoxification. We found α-esterases have experienced expansion in the common eastern firefly and large milkweed bug (Supplementary Table S3), however we did not detect convergent expansion of CYPs and GSTs in three CG-adapted insects. This may be due to high rates of gene birth and death dynamics in multiple insect lineages for some gene families including CYPs (Feyereisen, 2006; Sezutsu et al., 2013), and high error rates in true ortholog identification for large families in CAFE.

CGs are toxic and extremely unpalatable even at low concentration (Brower and Glazier, 1975; Agrawal et al., 2012). Most animals would avoid CG-rich food, however, herbivores specialized on CG-rich host plants probably evolve distinct preferences. We found four gene families expanded in two herbivorous monarch butterfly and large milkweed bug including CG5065 which has stable and essential core functions in fatty acid biosynthesis, and have no more than one copy in all surveyed 12 CG-sensitive insects and 12 *Drosophila* species (Finet et al., 2019). Moreover, insects have a variety of chemosensory gene families including gustatory receptors (GRs) (Eyun et al., 2017), and gene enrichment analyses of rapidly evolving gene families also reveals that chemosensory behavior (GO:0007635) and sensory perception of taste (GO:0050909) were among the most significantly enriched terms (Figure 1B; Supplementary Table S4). We found that a group of GRs has experienced expansion in the large milkweed bug and common eastern firefly (Supplementary Table S3). This GR is homologous to gustatory receptor 28b (*GR28b*) that plays a role in saponin detection in *D. melanogaster* (Sang et al., 2019). Saponin is a class of toxic chemical compounds found in many plants, and its structure is similar to CGs. The expansion of this GR family may be associated with its sensing and preference change to CGs in host *Asclepias* plants for large milkweed bug and conspecific recognition for common eastern firefly. However, we did not find significant changes in this GR family size in monarch butterfly, which suggests that monarch may use a different mechanism.

### Positive selection and convergent signals in CG-adapted insects

Herbivorous monarch butterfly and large milkweed bug can feed on and concentrate toxic cardiac glycosides, normally derived from their host plants, within various parts of the body or dorsolateral spaces, respectively (Brower and Glazier, 1975; Scudder and Meredith, 1982a; Bramer et al., 2016; Agrawal et al., 2023; Betz et al., 2025). Although storing CGs in different places, both monarch butterfly and large milkweed bug tend to select less toxic polar CGs as their defensive compound (Scudder and Meredith, 1982b; Bramer et al., 2016; Jones et al., 2019; Agrawal et al., 2023; Betz et al., 2025), suggesting that toxic CGs can also be an impossible burden on them. Indeed, convergent evolution in ATPα, the direct target of CGs, has been extensively studied (Zhen et al., 2012; Karageorgi et al., 2019; Taverner et al., 2019), and possible alternative mechanisms have also been proposed such as peritrophic membrane in the midgut and epithelial diffusion barriers (Barbehenn, 2001; Petschenka et al., 2013).

Here, we identified two septate junction-related genes *l(2)gl* and *crb* that are under positive selection in both monarch butterfly and large milkweed bug. In *Drosophila*, l (2)gl together with Dlg and Scribble (Scrib) proteins localize to the basolateral region of epithelial cells, while the apical domain is specified by cub, Stardust, and Par6/atypical protein kinase C (aPKC) complex, these compound play important roles in cell polarity maintance, junction formation and cell growth (Assémat et al., 2008; Yamanaka, 2008).

Furthermore, to detect genome-wide convergence signals among the three CG-adapted species, we employed identical methods for the convergence scan. Gene enrichment analyses using topGO also revealed that, among the top 20 terms, cell adhesion was significantly enriched in the CG-adapted species (Figure 2A; Supplementary Figure S2; Supplementary Table S8). We also used three additional methods to detect convergent signals (Supplementary methods and results), but we need to keep in mind that high false discovery rate of amino acid convergence due to stochastic neutral events and mutational constraints unrelated to the trait under convergence has been a huge concern (Zou and Zhang, 2015; He et al., 2020; Losos et al., 2020; Porter et al., 2023). Several studies found amino acid convergence in species with convergent phenotypes is not higher than the background and neutral expectations (Thomas and Hahn, 2015; Zhou et al., 2015; Davies et al., 2018). Additionally, convergent amino acid substitution is only one of many possible adaptive changes that may contribute to convergent phenotypes. Phenotypic convergence could also arise from changes at different sites, genes, and pathways, depending on the genetic architecture and evolutionary constraints of the trait. Nevertheless, scanning for molecular convergence in species with convergent phenotypes provides a useful approach to identify candidates underlying genetic basis of the focal phenotype for further investigation, and has great advantage when there is limited priori knowledge on the phenotype.

### Septate junctions that may contribute to CG adaptation by modulating epithelial permeability

Because the tissue-specific exposure to CGs, paralogs of ATPα that differs in activity and resistance against cardiac glycosides were evolved in insects (Zhen et al., 2012; Dalla et al., 2017; Lohr et al., 2017; Herbertz et al., 2023), and they show different distribution patterns and play different functional roles when in combination with different copies of ATPβ (Herbertz et al., 2022; Herbertz et al., 2023; Herbertz et al., 2025). Recent studies also found that in spite of functioning as enzymes maintaining membrane potentials, components of NKAs were found to colocalize with septate junction proteins such as *Nrx-IV*, neuroglian (*Nrg*), *Gli*, and Discs large (*Dlg*), hinting that NKAs are involved in septate junction formation and functionality (Genova and Fehon, 2003; Oshima and Fehon, 2011; Herbertz et al., 2025).

In invertebrate, septate junctions play barrier functions and localize in a variety of tissues, including malpighian tubules, guts and central nervous systems (Tepass and Hartenstein, 1994; Rouka et al., 2020; Jonusaite and Rodan, 2021; Contreras et al., 2024). We found that several highly conserved genes critical for septate junction integrity have convergent amino acid substitutions in CG-adapted species, including *Mcr*, *bark*, *Gli*, *Nrx-IV*, *zip* and *l(2)gl* (Supplementary Table S9). These genes play important roles in formation of a functional and highly crosslinked septate junction in invertebrate (Hindle and Bainton, 2014; Limmer et al., 2014). Among these genes, convergent signal of *Gli* only occurs in CG-adapted insects, and its role in septate junction is well studied. *Gli* is located at tricellular junctions and is necessary for septate junction development (Schulte et al., 2003), and mutations in *Gli* may result in delayed dorsal closure and incomplete peripheral blood-nerve barrier in peripheral glia in *D. melanogaster* embryos (Auld et al., 1995; Schulte et al., 2003). Interestingly, we identified a convergent amino acid substitution V581L in *Gli* in CG-adapted insects (Figures 2B,C). The convergent amino acid substitution might have functional consequences through septate junctions to the permeability of important barriers that relate to the CG-adaptation.

Consistent with our proposal, our studies show that the dihedral angles of the bilayer membranes near the tricellular junction are unequal and electron-dense material is absent at the center of the tricellular junction in the *Gli* mutant (Figure 4). Similar abnormalities in the TCJ were observed in aka mutant *Drosophila* embryos, which will lead to the defective epithelial barrier function of hindgut, salivary glands, and tracheae in mutant embryos (Byri et al., 2015). Our finding that the *Gli* V-to-L substitution reduces survival in *Drosophila* may initially appear contradictory to its role as an adaptive mechanism in nature. The valine residue at this position is highly conserved across insects, suggesting it is critical for the native physiological function of *Gli*, potentially in maintaining epithelial integrity. In naturally CG-adapted lineages, the beneficial effect of this substitution (conferring toxin resistance) is likely preserved while its deleterious side effects are mitigated through compensatory evolution. This could involve: secondary substitutions within the Gli protein itself that restore structural stability; co-evolution of interacting partners (e.g., components of the septate junction or ATPα); upregulation of compensatory pathways (such as the cell adhesion or detoxification genes identified in our convergent analyses). In our experimental system, the *Gli* mutant carries the adaptive substitution but lacks this broader co-adaptive genetic background.

Beyond, we also proposed that this disruption may facilitate the sequestration of toxic substances. Indeed, in the mosquito (*Aedes aegypti*), rearing their larvae in seawater will cause an increase in Gli protein abundance in the anterior midgut, malpighian tubules and hindgut, and knockdown of *Gli* in the midgut increased the flux rate of the paracellular permeability marker (Jonusaite et al., 2017). For CG-adapted large milkweed bug, previous studies show that compared to locusts (*Schitocerca gregaria*) and cockroaches (*Periplaneta americana*), only midgut of the large milkweed bug is permeable to CGs, and polar ouabain without being metabolized can directly cross the midgut, sequestered from the haemolymph into the dorsolateral spaces (Scudder and Meredith, 1982b). To balance the requirements of CG resistance and effective ion transport, the nervous tissue of the large milkweed bug predominantly expresses the highly CG-resistant but less active α1B subunit and the highly active but less resistant α1C subunit. These subunits co-occur with β2 and β3 subunits, forming larger complexes that extend beyond simple heterodimers (Herbertz et al., 2022). The resistant NKA of milkweed bugs is selected for working optimally in a “toxic environment,” that is, when sequestered cardenolides are stored in the body (Pokharel et al., 2021). While for the monarch butterfly, another CG-adapted insect, the uptake of CGs by the intestinal epithelium is also a very rapid process (Betz et al., 2025), but we did not detect the convergent amino acid substitution in the *Gli* gene, additional mechanisms such as metabolism or excretion may be involved.

## Data Availability

The original contributions presented in the study are included in the article/Supplementary Material. All public datasets used in this analysis are summarized in Supplementary Table S1, which includes accession numbers and source databases. The custom code used for analysis is available at GitHub: https://github.com/kanglizhu/convergent-analyses-in-insects. Further inquiries can be directed to the corresponding author.
